# Construction of Graphite Shells on Ferromanganese Oxide for Electromagnetic Wave Absorption

**DOI:** 10.3390/ma18235336

**Published:** 2025-11-26

**Authors:** Yuxiang Zhang, Shuling Shen, Jing Li

**Affiliations:** School of Materials and Chemistry, University of Shanghai for Science and Technology, No. 516 Jungong Road, Shanghai 200093, China

**Keywords:** electromagnetic wave absorption, core–shell, reflection loss, impedance matching

## Abstract

Ferromanganese oxide (FMO), a by-product of steelmaking industry, was coated with polyacrylonitrile (PAN) to construct an electromagnetic wave absorber (FMO@C) with a core–shell structure. The effect of heat treatment from 600 to 1000 °C on the phase transformation of FMO and carbonization of PAN was studied. Upon the heat treatment at 1000 °C, the reflection loss and effective bandwidth of the FMO@C reached −18.20 dB and 3.08 GHz at a thickness of 1.6 mm, presenting a significant improvement over FMO which only exhibited a reflection loss of −2.31 dB at 10 mm. Boric acid was infiltrated into the PAN shells to catalyze the carbonization process and adjust the impedance matching, which further improved the reflection loss to a minimum value of −28.25 dB. Via varying the concentration of boric acid, the reflection loss of −22.01 dB with an effective bandwidth of 3.36 GHz at a thickness of 1.3 mm was achieved. The enhanced EMW absorption performance was attributed to multiple reflections and polarization caused by the core–shell structure, magnetic loss from the phase transformation of FMO, dielectric loss from carbon shells, as well as the tunable impedance matching by boron-catalyzed carbonization. The construction of the core–shell structure could be a promising downstream processing of FMO and could extend the application of the solid wastes.

## 1. Introduction

Electromagnetic waves (EMW) are essential to modern society, yet they pose risks such as harmful interference to electronic devices and potential health hazards from prolonged exposure [1,2]. EMW-absorbing materials are widely needed for human health, equipment shields, and information security [3].

Industrial wastes from steelmaking processes, including iron powders, iron oxide scales, and steel slag, possess excellent magnetic properties [4,5]. Research attempts have been made to utilize these industrial solid wastes as EMW absorbers. Steel slag was added in an EMW absorption mortar, which exhibited a minimum reflection loss (RL) of −11.5 dB at a thickness of 25 mm [6]. Cement-based composites were reinforced by waste iron powders and the minimum RL was −12.78 dB with an effective absorption bandwidth of 1.37 GHz at a thickness of 3.55 mm [7]. In addition, iron oxide scales were also used as EMW-shielding materials [4,8,9]. Ozturk et al. produced an EMW-shielding mortar containing 30 vol% of iron oxide scales, which exhibited a shielding effectiveness of approximately 40 dB at a thickness of 20 mm in the 11–18 GHz frequency range [4]. Adam Jakubas et al. prepared EMW-shielding materials by hot pressing recycled high-density polyethylene (HDPE) and iron oxide scales. The composites containing 70 wt% iron oxide scale in the HDPE matrix exhibited an electromagnetic shielding effectiveness of up to 52 dB in the 8.1–12.1 GHz range [8]. Nevertheless, the application of industrial solid wastes is limited due to their high content of impurity, which may result in unstable electromagnetic properties. Ferromanganese oxide (FMO) is a secondary processing product made from the recycled iron oxide scales, which opens an opportunity to produce low-cost EMW-absorbing materials in large quantity. Compared with iron oxide scales, the industrial-grade pre-sintered FMO powder, consisted of Mn_2_O_3_ and Fe_2_O_3_, have a well-defined composition and obvious magnetic characteristics. The addition of manganese oxide can generate interface inhomogeneity and interface polarization loss of EMW [10,11].

The structural design of carbon/magnetic composites is vital for optimizing their EMW absorption performance. Core–shell structures, skeleton structures, and hollow structures were studied, and all these structures enhanced EMW absorption through distinct mechanisms [12]. The skeleton structure employed a three-dimensional conductive network, which was lightweight and which promoted multiple scattering [12]. The hollow structure created internal cavities to enhance multiple reflections of EMW [13]. The core–shell structure was acknowledged as one of the most favourable structures for EMW absorption, as it can combine multi-function materials, protect the core materials, and facilitate interfacial polarization and multireflection [14,15,16]. Porous carbon-coated CoFe_2_O_4_ exhibited a minimum RL of −39.48 dB at 1.22 GHz with a 2 mm thickness [17]. BaFe_12_O_19_ was coated by glucose-based carbon, achieving an optimal RL of −73.42 dB at 17.84 GHz with a thickness of 1.40 mm [18]. MnO_2_-coated Fe_3_O_4_ microspheres showed an optimal RL of −48.5 dB at 11.2 GHz with a thickness of 2.5 mm [19]. Pyrolyzed carbon-coated Co had an optimal RL of −96.2 dB at 5.8 GHz with a thickness of 3.1 mm [20]. The high EMW absorption properties in these works are benefited from the core–shell structure with nano-sized or submicron-sized magnetic cores.

The doping of foreign elements on carbon materials can catalyze their graphitization process [21], improve their electrical conductivity, and facilitate the EMW absorption [22]. Boron has a similar atomic radius and less electronegativity than carbon, so it can enter the carbon lattice at substitutional position and function as a p-type dopant, which introduce additional defects, improves impedance matching, and enhances polarization loss, conductive loss and dielectric loss [22]. Immersion in boric acid, followed by heat treatment, was reported as a facile way to dope carbon fibres and catalyze their graphitization, which improved significantly the electrical and thermal conductivity of carbon fibres [21,23].

Aiming for an extended utilization of industrial solid wastes, a series of magnetic core@carbon shell (FMO@C) materials was constructed. Polyacrylonitrile (PAN) was pyrolyzed to produce the carbon shells. The effect of heat treatment was studied on the phase evolution of FMO and the carbonization of PAN. The boric acid was used to catalyze the carbonization and tune the impedance matching. The EMW-absorbing properties were analyzed based on the dielectric loss, magnetic loss, and the impedance matching characteristics. Eventually, the RL values of FMO@C reached −28.25 dB, exhibiting a much higher EMW absorption capability than FMO, owning to the controlled phase transformation of core materials and the catalyzed graphitization of carbon shells.

## 2. Materials and Methods

### 2.1. Fabrication of the FMO@C Powder

FMO was supplied by Shanghai Baosteel Magnetic Materials Co., Ltd. (Shanghai, China) PAN (average M.W. = 150,000, and was dissolved in N, N-dimethylformamide (DMF) at a concentration of 4.3%. FMO was then dispersed in DMF with a content of 10 wt% by sonicator for 10 min. After the evaporation of DMF at 120 °C, the mixture was calcined at 270 °C for 120 min for the cyclization of PAN (referred as FMO@PAN-270). Then, the products were carbonized in a tubular furnace under N_2_ atmosphere at different temperatures (600, 700, 800, 900, and 1000 °C) for 2 h. The FMO@C were denoted as FMO@C-600, FMO@C-700, FMO@C-800, FMO@C-900, and FMO@C-1000, according to the carbonization temperature. Alternatively, FMO@PAN-270 was dipped into a boron acid (H_3_BO_3_) solution of different concentrations (0.5%, 1%, and 2.5%) for 6 h at 80 °C before the carbonization process at 1000 °C. The samples were named as FMO@C-B1, FMO@C-B2, and FMO@C-B3, respectively.

### 2.2. Characterization

The morphological evolution and phase transition were tested by a scanning electron microscope (Thermo Apreo 2S HiVac, Thermo Fisher Scientific, Waltham, MA, USA), X-ray diffractometer (D8 ADVANCE, Bruker, Billerica, MA, USA), and Fourier transform infrared spectrometer (FT-IR, SPE CTRUM 100, PerkinElmer, Waltham, MA, USA). The graphitization degree was characterized by Raman spectroscopy (HORIBA, Kyoto, Japan) equipped with a 532 nm laser. The electrical conductivity of FMO@C was measured by the four-probe technique (SB120/2, China). The electromagnetic properties were evaluated using a vector network (Agilent N5244A, USA) across the 2–18 GHz frequency band. The FMO@C specimens were homogeneously blended with paraffin at a 1:1 weight ratio and compacted into a cylindrical mould (Φout = 7.00 mm, Φin = 3.00 mm).

## 3. Results and Discussion

### 3.1. The Phase Evolution of FMO@C

**Figure 1:**
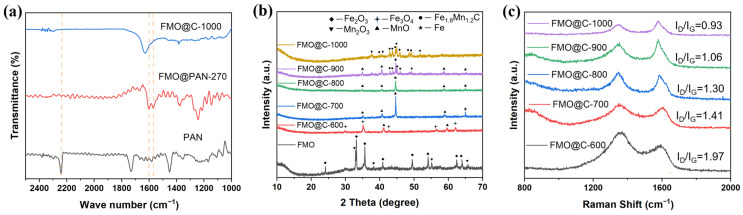
(**a**) FT-IR curves of PAN, FMO@PAN-270 and FMO@C-1000, (**b**) XRD patterns of FMO and FMO@C, and (**c**) Raman spectrum of FMO@C.

The FT-IR spectrum of PAN had a characteristic peak at 2245 cm^−1^, as shown in Figure 1a, which was attributable to -C≡N tensile vibration [24]. Upon cyclization at 270 °C, new peaks at 1603 cm^−1^, 1587 cm^−1^, and 1369 cm^−1^ were ascribed to -C=N, -C=C, and -C-H bonds, respectively, as a result of the typical transformation of PAN from a linear chain to a cyclized structure [25]. Upon carbonization at 1000 °C, the majority of the peaks were diminished, suggesting the pyrolysis process. A new peak at 1632 cm^−1^ was denoted to C=C or C=N conjugated bonds in aromatic rings [26].

The XRD spectra of FMO@C samples are presented in Figure 1b. The as-received FMO were composed of Fe_2_O_3_ and Mn_2_O_3_, with diffraction peaks corresponding to (JCPDS No. 33-0664) and (JCPDS No. 89-4836). Fe_3_O_4_ phase (JCPDS No. 19-0629) and MnO phase (JCPDS No. 07-0230) were formed in FMO@C-600 by reduction at 600 °C. XRD spectrum of the FMO@C-700 and FMO@C-800 samples showed mainly Fe upon further reduction. For FMO@C-900, the characteristic peaks of Fe_1.8_Mn_1.2_C (JCPDS No. 89-2546) appeared along with Fe and MnO phases. After the heat treatment at 1000 °C, the peaks corresponding to Fe and MnO disappeared, leaving only the Fe_1.8_Mn_1.2_C phase, which remained stable when the heating temperature was further increased to 1200 °C. The phase transformation played a fundamental role in regulating the final electromagnetic properties of the core–shell materials.

The graphitization degree of the shells can be analyzed by Raman spectra, as shown in Figure 1c. The G band around 1580 cm^−1^ arises from the stretching motions of ordered sp^2^ carbon bonds, whereas the D band near 1350 cm^−1^ comes from disordered breathing modes caused by structural imperfections [27]. The intensity of the ratio of the D to G bands (I_D_/I_G_) of the shells decreased continuously with increases in the heat treatment temperature, which suggests an increased degree of graphitization [28].

**Figure 2:**
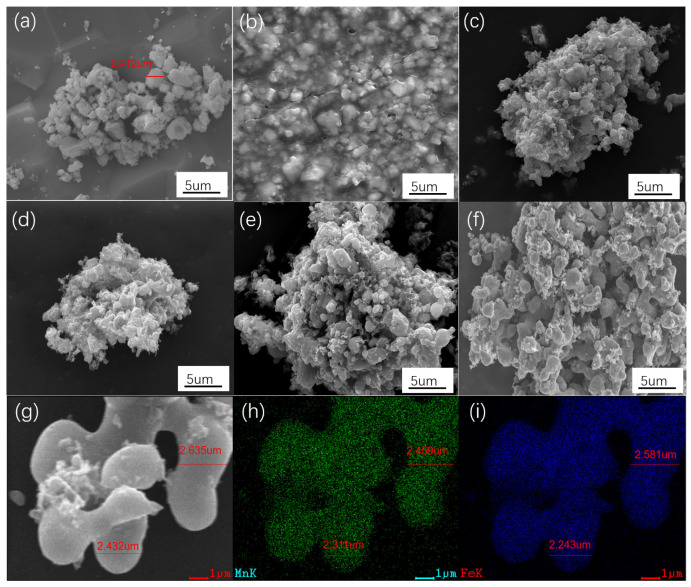
The SEM images of (**a**) FMO, (**b**) FMO@PAN, (**c**) FMO@C-700, (**d**) FMO@C-800, (**e**) FMO@C-900, (**f**) FMO@C-1000 (low magnification), (**g**) FMO@C-1000 (high magnification), and the EDS (**h**) Mn and (**i**) Fe element mappings.

The as-received FMO particles had a diameter of 1–3 μm (Figure 2a). The PAN-wrapped FMO particles sticked together, forming large aggregates, as shown in Figure 2b. The polymetric morphology gradually disappeared upon the heat treatment (Figure 2c–e). Eventually, when the heating temperature reached 1000 °C, the aggregates were separated into core–shell particles because the carbonization of the PAN caused a volume shrinkage during the structural evolution of the shells, as shown in Figure 2f. With the higher magnification seen in Figure 2g, interconnected FMO@C-1000 particles were observed with a size of around 2 μm, similar with that of the as-received FMO. EDS Mn and Fe element mappings confirmed the wrapping of FMO in the particles as shown in Figure 2h,i. Along with the typical Raman spectra of carbon in Figure 1c, the core–shell structure can be proven.

### 3.2. EMW Absorption Properties of FMO@C

To investigate the EMW absorption properties of FMO@C, the RL values were calculated using the equations shown below [29]:

(1) $$RL=20\log\left|(Z_{in\;}-Z_{0})/(Z_{in\;}+Z_{0})\right|$$

(2) $$Z_{in}=Z_{0}{\left(\mu_{r}/\varepsilon_{r}\right)}^{1/2}\tanh\;\left(j(2\pi fd/c\right){\left(\mu_{r}\varepsilon_{r}\right)}^{1/2}$$

where $Z_{in}$ and $Z_{0}$ are the input impedance of the absorber and the impedance of the free space. The f is the EMW frequency, h is the Planck constant, c is the light velocity, and d refers to the absorber thickness. The effective absorption bandwidth refers to the portion where RL is below −10 dB [30].

**Figure 3:**
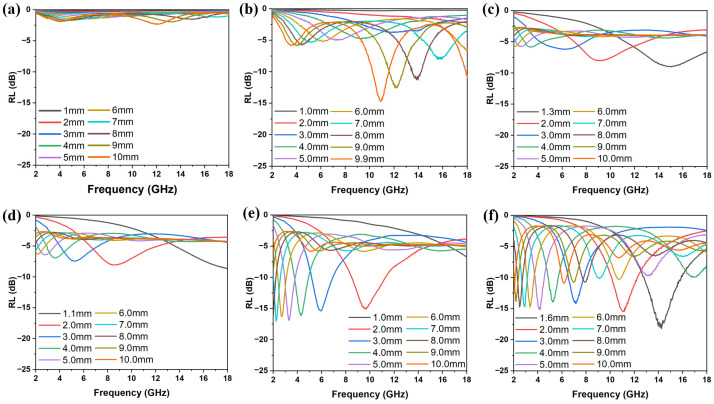
The RL curves with different thicknesses in the frequency range of 2–18 GHz: (**a**) FMO, (**b**) FMO@C-600, (**c**) FMO@C-700, (**d**) FMO@C-800, (**e**) FMO@C-900, and (**f**) FMO@C-1000.

The RL values of FMO@C are shown in Figure 3. The pristine FMO had a virtually negligible capability of EMW absorption with a minimal RL of −2.31 dB at a thickness of 10.00 mm. As shown in Figure 3b–f, the FMO@C-600, FMO@C-900, and FMO@C-1000 showed a much higher EMW absorption capacity than FMO, FMO@C-700, and FMO@C-800. FMO@C-1000 had the minimum RL value of −18.20 dB with a thickness of 1.6 mm, and the effective bandwidth was 3.08 GHz. The minimum RL values of FMO@C-700 and FMO@C-800 were only −9.04 dB and −8.67 dB, respectively.

**Figure 4:**
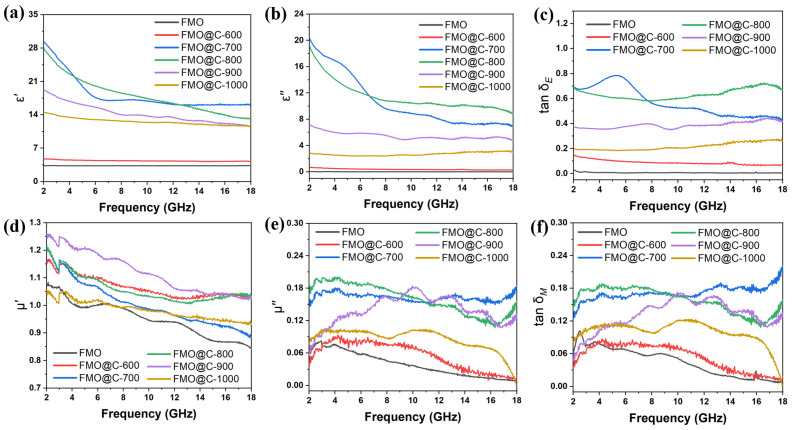
Dielectric constant and permeability of FMO@C heated at different temperatures: (**a**) real part of permittivity, (**b**) imaginary part of permittivity, (**c**) the dielectric loss tangent, (**d**) real part of permeability, (**e**) imaginary part of permeability, and (**f**) the magnetic loss tangent.

To acquire a more profound understanding of the mechanisms underlying EMW absorption properties, the electromagnetic parameters of FMO@C were investigated. The real and imaginary parts of the complex dielectric constant $\left(\varepsilon_{r}=\varepsilon^{\prime }-j\varepsilon^{\prime \prime }\right)$ and complex magnetic permeability $\left(\mu_{r}=\mu^{\prime }-j\mu^{\prime \prime }\right)$ are shown in Figure 4a,b,d,e. The real parts (ε′ and μ′) represent the energy storage capacity within the EMW absorber, while the imaginary parts (ε″ and μ″) characterize its dissipative properties [31]. The ε′ and ε″ values of FMO were relatively low, suggesting that the dielectric storage and dissipation capabilities were weak. The ε′ and ε″ values of FMO@C-600 surpassed FMO, mainly due to the construction of the carbon shells. Moreover, the values of ε′ and ε″ of FMO@C-700 and FMO@C-800 significantly increased, caused by the transformation of Fe_3_O_4_ into Fe [32]. As the carbonization temperature was higher than 900 °C, the dielectric properties of FMO@C progressively decreased as the Fe and MnO converted to Fe_1.8_Mn_1.2_C.

**Figure 5:**
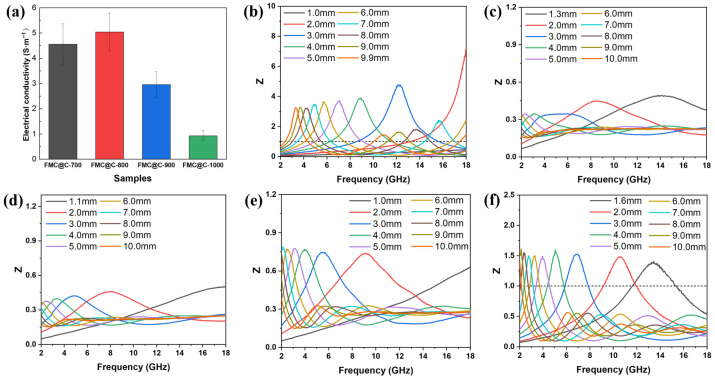
(**a**) Electrical conductivity of FMO@C, the impedance matching of (**b**) FMO@C-600, (**c**) FMO@C-700, (**d**) FMO@C-FMO@C-800, (**e**) FMO@C-900, and (**f**) FMO@C-1000.

The electrical conductivity of the samples are shown in Figure 5a. According to the equation derived from the free electron theory ($\varepsilon^{\prime \prime }\approx \sigma /2\pi \varepsilon_{0}f$, where σ represents the conductivity of materials), the conductivity is directly proportional to the value of ε″ [33]. The electrical conductivity of FMO and FMO@C-600 was lower than the testing limitation. The electrical conductivity of FMO@C-700 and FMO@C-800 was higher than that of FMO@C-900 and FMO@C-1000, exhibiting the same trend with ε″ values. The dielectric loss (tan δ_E_) of the FMO@C was calculated by $\tan\delta_{E}{=\varepsilon^{\prime \prime }/\varepsilon }^{\prime }$ and shown in Figure 4c. FMO@C-700 and FMO@C-800 possessed the highest tan δ_E_ values, indicating that they possessed the strongest dielectric loss capabilities.

Figure 4d–f show the μ′, μ″, and tan δ_M_ of FMO@C. The FMO@C-700 and FMO@C-800 exhibit higher tan δ_E_ and tan δ_M_ than other samples. However, their RL was not comparable to the other FMO@C specimens. Impedance matching $\left(Z=\left|Z_{in}/Z_{0}\right|\right)$ is another crucial parameter beyond the loss capability of materials. When the Z values of material are equal to 1, EMW will fully enter the material without reflection [34]. The Z values of FMO@C-900 and FMO@C-1000 were closer to 1, as shown in Figure 5, granting them higher RL values, even though their tan δ_E_ and tan δ_M_ values were not the highest. In contrast, FMO@C-700 and FMO@C-800 exhibited Z values significantly lower than one because the presence of elemental Fe resulted in their high dielectric constants and high electrical conductivity [34].

### 3.3. Effects of Boric Acid Catalyzation

To further enhance the EMW absorption properties of FMO@C, the graphitization process of the carbon shells was catalyzed by boric acid. At approximately 220 °C, boric acid underwent dehydration and decomposed into boron oxide (B_2_O_3_) [23]. As the temperature further increased, B_2_O_3_ reacted with carbon, playing a role in promoting graphitization [35].

**Figure 6:**
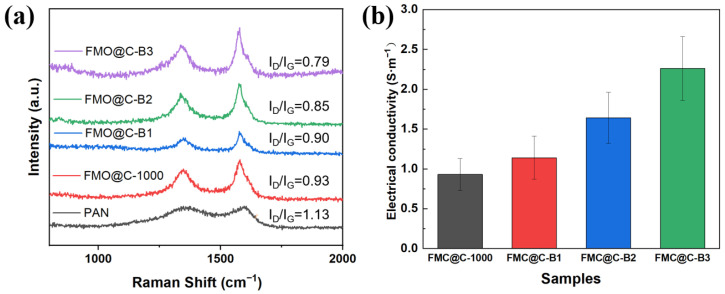
(**a**) Raman spectrum and (**b**) electrical conductivity of PAN-based carbon, FMO@C-1000, and FMO@C-B.

Figure 6 shows the Raman spectra and electrical conductivity of samples. The I_D_/I_G_ values of FMO@C-1000 were lower than that of pure PAN-based carbon after carbonization at 1000 °C, suggesting that FMO and its reduction products can facilitate the graphitization of carbon materials. The iron oxides or elemental Fe acted as catalysts that promoted the rearrangement of amorphous carbon into ordered graphitic layers through a dissolution–precipitation process [36,37]. The I_D_/I_G_ of FMO@C gradually decreased as increasing the boric acid concentration, leading to the increasing of their electrical conductivity due to the enhanced graphitization degree of the carbon shells.

**Figure 7:**
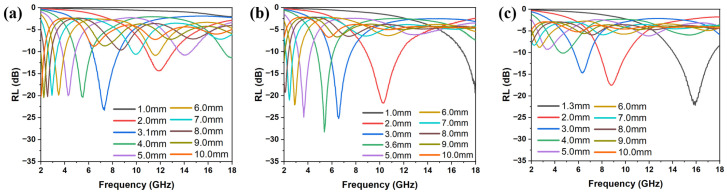
The RL curves of (**a**) FMO@C-B1, (**b**) FMO@C-B2, and (**c**) FMO@C-B3.

The FMO@C-B1 exhibited a RL value of −23.11 dB and effective bandwidth of 2.24 GHz with a thickness of 3.1 mm, as shown in Figure 7. The FMO@C-B2 achieved the best RL of −28.25 dB and effective bandwidth of 1.44 GHz with a thickness of 3.6 mm, while it had a RL value of −21.68 dB with an effective bandwidth of 2.92 GHz at a thickness of 2.0 mm. The RL of FMO@C-B3 was decreased to −22.01 dB, along with the widest effective bandwidth of 3.36 GHz at a thin thickness of 1.3 mm. All of the FMO@C-B specimens showed better EMW absorption properties than the specimens without the treatment by boric acid.

**Figure 8:**
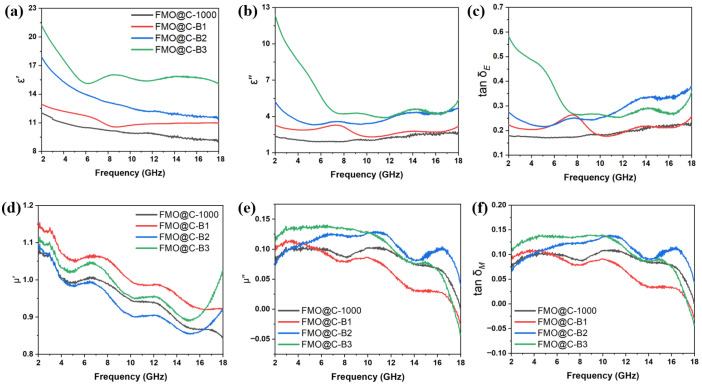
Dielectric constant and permeability of FMO@C-B: (**a**) real part of permittivity, (**b**) imaginary part of permittivity, (**c**) the dielectric loss tangent, (**d**) real part of permeability, (**e**) imaginary part of permeability, and (**f**) the magnetic loss tangent.

The ε′, ε″, and tan δ_E_ of FMO@C-B are shown in Figure 8a–c. The dielectric constant increased with the degree of graphitization. The dielectric constant of FMO@C-B was generally higher than those of FMO@C. The tan δ_E_ curves of all FMO@C-B exhibit multiple resonance peaks, indicating the presence of multiple polarization relaxations, which were beneficial for dielectric loss [20].

Figure 8d–f show the μ′, μ″, and tan δ_M_ of the FMO@C-B. The tan δ_E_ values of FMO@C were significantly higher than the tan δ_M_ values, indicating that dielectric loss played a primary role in the attenuation of EMW energy. All samples exhibited similar trends in tan δ_M_ curves and displayed resonance peaks at 9–12 GHz and 15–18 GHz, which may be related to natural resonance and exchange resonance [20].

The attenuation constant of FMO@C-1000 and FMO@C-B was calculated using the following equation [38]:

(3) $$\alpha =\frac{\sqrt{2}}{c}\pi f\times \sqrt{\left(\mu^{\prime \prime }\varepsilon^{\prime \prime }-\mu^{\prime }\varepsilon^{\prime }\right)+\sqrt{{\left(\mu^{\prime \prime }\varepsilon^{\prime \prime }-\mu^{\prime }\varepsilon^{\prime }\right)}^{2}+{\left(\mu^{\prime }\varepsilon^{\prime \prime }+\mu^{\prime \prime }\varepsilon^{\prime }\right)}^{2}}}$$

**Figure 9:**
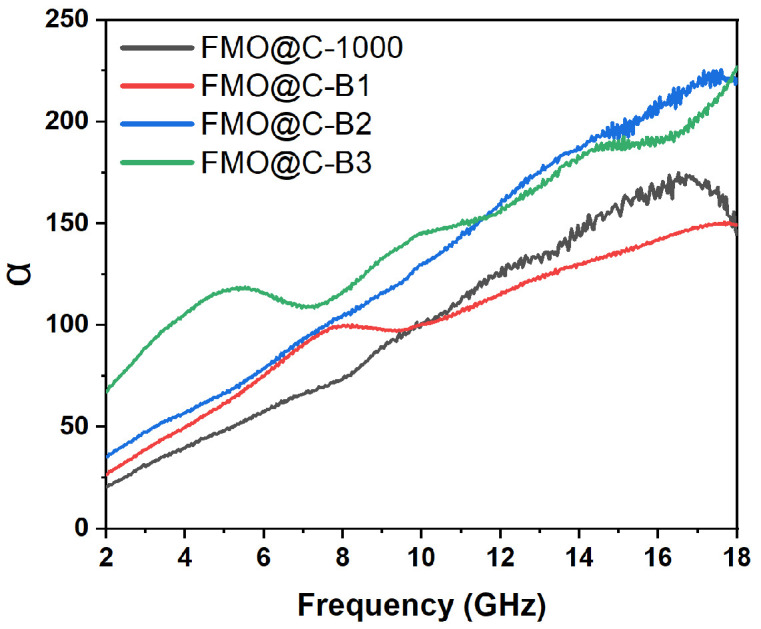
The values of the attenuation constant for FMO@-1000 and FMO@C-B in the frequency range of 2.0–18.0 GHz.

**Figure 10:**
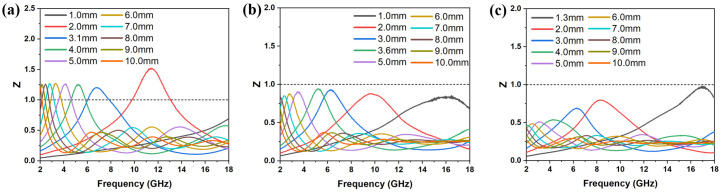
The impedance matching of (**a**) FMO@C-B1, (**b**) FMO@C-B2, and (**c**) FMO@C-B3.

As shown in Figure 9, FMO@C-B2 and FMO@C-B3 exhibited higher attenuation constants than FMO@C-B1 and FMO@C-1000, indicating their stronger attenuation capabilities primarily attributed to their superior dielectric loss. Figure 10 illustrates the impedance matching of FMO@C-B specimens. It seems that the FMO@C-B2 had Z values nearer to one compared with the other samples. The attenuation ability and impedance matching of FMO@C-B2 were in agreement with its excellent RL value and explained the effects of boric acid on the EMW absorption properties.

**Figure 11:**
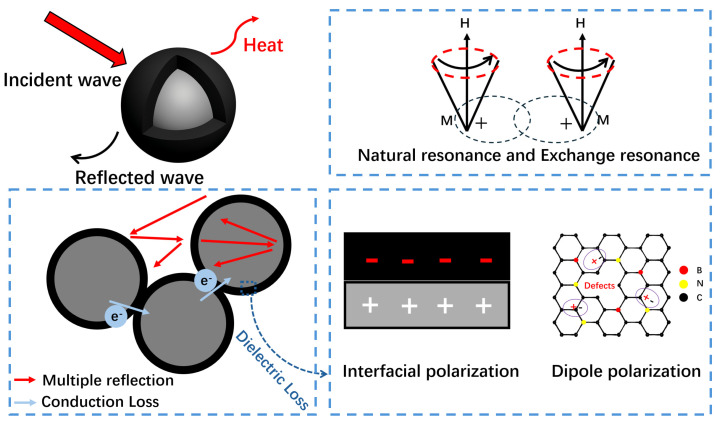
Schematic illustration of EMW absorption mechanism of FMO@C-B.

The EMW absorption mechanisms of FMO@C-B are schematically presented in Figure 11. Firstly, the core–shell structure of the material facilitated multiple reflections of EMW at the interfaces. Meanwhile, the carbon shells of the particles were interconnected rather than isolated, which promoted the electron transportation between particles and enhanced the conductive loss of materials. Secondly, heterogeneous interfaces within the materials induced interfacial polarization, while the lattice defects and amorphous carbon structure in the carbon shells generated dipole polarization. These polarization relaxation processes amplified the dielectric loss of the materials. Furthermore, the magnetic cores generated multiple magnetic resonances, including natural resonance and exchange resonance. The dielectric and magnetic losses improved the material’s EMW absorption performance. Lastly, the impedance matching between magnetic cores and the carbon shells was optimized through the catalyzed graphitization of the carbon shells.

## 4. Conclusions

FMO@C with a core–shell structure was constructed using PAN-wrapped FMO as a precursor, aiming for the extended utilization of the industrial solid waste. When the heating temperature ranged from 600 to 1000 °C, FMO was transferred into different phases with varying dielectric and magnetic properties, and the graphitization degree of the carbon shells also changed. The FMO@C-1000 had a minimum RL of −18.20 dB and an effective bandwidth of 3.08 GHz at only a 1.6 mm thickness, which was superior to as-received FMO. Boric acid-catalyzed graphitization of carbon shells further enhanced the RL to a minimum value of −28.25 dB with an effective bandwidth of 1.44 GHz at a thickness of 3.6 mm because of the increased dielectric loss and tuned impedance matching. The RL of −22.01 dB with larger effective bandwidth of 3.36 GHz at smaller thickness of 1.3 mm was achieved by varying the concentration of boric acid. The FMO@C could be cost-efficient EMW absorbers and could be applied as fillers in composites for EMW-proof packaging or building. The core–shell construction, as a downstream processing of FMO, was simple and would be easy to upscale.

## Data Availability

The original contributions presented in this study are included in the article. Further inquiries can be directed to the corresponding authors.
